# Relationship between Religious Coping, Pain Severity, and Childbirth Self-Efficacy in Iranian Primipara Women

**DOI:** 10.1155/2022/2338683

**Published:** 2022-02-15

**Authors:** Tahereh Sotudeh, Seyedeh Batool Hasanpoor-Azghady, Leila Amiri-Farahani

**Affiliations:** Department of Midwifery and Reproductive, School of Nursing and Midwifery, Iran University of Medical Sciences, Tehran, Iran

## Abstract

**Background:**

One of the important goals of midwifery support and care is to control labor pain and increase the ability to cope with pain. The use of religious coping may be effective in counteracting the stressors of labor, especially labor pain, as well as increasing the self-efficacy of labor. This study was conducted to determine the relationship between religious coping, pain severity, and childbirth self-efficacy in Iranian primipara women.

**Materials and Methods:**

This cross-sectional study was performed on 200 Iranian primiparous women referred to eight health centers in the capital of Hormozgan Province who were intending to have a normal vaginal delivery (NVD) in the Persian Gulf and Sharifi Hospitals. The sampling was multistage. Data were collected by demographic and fertility questionnaires, the Iranian Religious Coping Scale, the Childbirth Self-Efficacy Inventory, and the Visual Analog Scale for pain measurement.

**Results:**

Among the dimensions of religious coping, benevolent reappraisal had a significant direct relationship with pain severity, and negative religious coping had a significant inverse relationship with pain severity. In the case of childbirth self-efficacy subscales, the results showed dimensions of religious practices, benevolent reappraisal, and active religious coping had a significant direct relationship with outcome expectancy, and negative religious coping had a significant indirect relationship with outcome expectancy. Also, there was a significant direct relationship between religious practices and efficacy expectancy and a significant inverse relationship between negative and passive religious coping and efficacy expectancy.

**Conclusion:**

With increasing some dimensions of positive religious coping, the severity of labor pain and childbirth self-efficacy increases, and with increasing dimensions of negative and passive religious coping, childbirth self-efficacy decreases. These correlations were weak in all the mentioned results.

## 1. Introduction

Childbirth makes women take on a parental role [1, 2], but experiencing labor pain is a very stressful event [3, 4]. Childbirth pain is one of the most severe pains women experience during their lives [5–7]. About 60% of women report labor pain as one of the most severe pains they have ever experienced [8]. Therefore, childbirth as the termination of a pregnancy is an important experience [9, 10]. High stress and pain during labor cause the fear of pregnancy and subsequent normal virginal delivery (NVD) [8]. For this reason, one of the major goals of midwifery support and care is to cope with labor pain [11]. Coping is a dynamic process of cognitive and behavioral responses to control and manage situations that are considered stressful. Different pharmacological and nonpharmacological methods are used to deal with labor pain. Nonpharmacological coping includes physiological coping (such as breathing techniques, relaxation, postural changes, and move during the labor), psychological coping (including social support, increasing self-efficacy, and increasing self-confidence), cognitive coping (including distraction, Illustration, and focus), and religious coping [12]. Religious coping reflects an individual's efforts to use religious teachings to understand and cope with life's stressful events and experiences [13]. Religion plays an integral role in dealing with stressful events. Pargament believes that religion is part of the process of coping because religion provides resources for people to deal with situations and helps them to evaluate situations differently, which increases their ability to deal with these situations [14, 15]. In general, religious and spiritual interpretations of life's painful events are those that increase a person's ability to face life's problems and obstacles [16]. Of course, religion and spirituality are not the same things, nor are they entirely distinct from one another [17]. Religion is a specific set of organized beliefs and practices, usually shared by a community or group. But spirituality is a broad concept with room for many perspectives. In general, spirituality includes a sense of connection to something bigger than ourselves, and it typically involves a search for meaning in life. However, spirituality may incorporate elements of religion, but it is generally a broader concept [18].

Although religious coping has a positive implication, religious coping is expressed in two ways: positive and negative religious coping. Positive religious coping shows a secure relationship with the transcendent force, a sense of connection to spirituality and others, and a benevolent view of the world [19]. In contrast, negative religious coping is a less secure relationship with God and a tenuous and sinister view of the world. This pattern includes a very different set of religious coping methods such as punitive religious reappraisals, demonic religious reappraisals, reappraisals of God's powers, spiritual discontent, self-directing religious coping, and interpersonal religious discontent [20]. For this reason, positive religious confrontation is associated with consequences such as self-esteem and higher quality of life and psychological adjustment, while negative religious confrontation is associated with consequences such as depression, mental disorder, poor physical health and quality of life, and poor problem solving [21].

Studies have shown that religious coping gives pregnant women the ability and confidence to cope with a pleasant delivery [22, 23]. Some people consider the difficult and exhausting moments of labor and childbirth to be spiritual, calling each birth a spiritual experience [24]. In Christianity, women are assisted through religious coping during labor and childbirth [24, 25]. Some Muslim women also read verses of the Quran for safe childbirth delivery [24]. Thus, in clinical settings, a religious perception of childbirth is essential [26].

Self-efficacy is one of the predictors of behavior during pregnancy and childbirth. According to Bandura's theory of self-efficacy, the experience of childbirth delivery is one of the most important factors associated with women's self-efficacy [27]. According to studies, religious coping also affects self-efficacy [17, 28] and may increase childbirth self-efficacy [29]. Callister cited childbirth as an opportunity to draw closer to God, as well as an opportunity to make religiosity more meaningful and the importance of supreme power in influencing birth and childbirth outcomes and a spiritual experience. He described the use of religious beliefs and rituals as powerful coping mechanisms [26]. The results of a study point to the role of religious coping styles such as prayer and relying on God in coping with labor pain [30] because people believe that God will not leave them alone in the face of a painful event. However, in negative religious coping, one establishes an avoidant and insecure relationship with God. For example, he believes that God will leave him alone in difficult moments [31, 32].

In recent years, domestic and foreign studies have been conducted to relieve pain and examine women's experiences of labor pain, but less attention has been paid to religious coping. Given the importance of religion in creating positive experiences, feelings of security, and self-efficacy in women [33], also considering the importance of religious belief in coping with pain and labor self-efficacy, this study aimed to determine the relationship between religious coping, pain severity, and childbirth self-efficacy in Iranian primiparous women.

## 2. Materials and Methods

This cross-sectional study was performed on 200 primiparous women referred to eight health centers in the capital of Hormozgan Province, Iran. All of the health centers were affiliated with Hormozgan University of Medical Sciences, Iran. The capital of Hormozgan Province is geographically divided into two areas: east and west. Each area was considered a stratum. The east area had 13 and the west area had 12 health centers. Four health centers were randomly selected from each area. The sample size was estimated at 194 subjects at a 95% confidence interval and 80% test power considering a Pearson correlation coefficient of 0.2 between religious coping, pain severity, and childbirth self-efficacy through the following formula: *n*=((*z*_1−(*α*/2)_+*z*_1−*β*_)^2^/*ω*^2^)+3; *ω*=(1/2)ln(1+*r*/(1 − *r*)).

Because the eligible samples selected at the health centers may not be eligible at the time of referral to the hospitals for delivery, 223 primiparous pregnant women were selected from the health centers, taking into account the 15% sample drop. The quota sampling was performed by the relative allocation method from May 2018 to July 2019. Inclusion criteria at the time of sampling in health centers included being 18–35 years old pregnant women, having the ability to read and write, receiving prenatal care, having a low-risk pregnancy, and intending to have a normal vaginal delivery (NVD) in the Persian Gulf and Sharifi Hospitals (the largest government hospitals located in the capital of Hormozgan Province). Inclusion criteria at the time of referral to the hospitals for delivery included having a gestational age of 37 to 42 weeks based on the last menstrual period (LMP) or first-trimester ultrasound, an estimated fetal weight of between 2500–4000 grams according to the ultrasound report, mother's admission at the beginning of the active phase based on the vaginal examination performed by the researcher, and not having a high-risk pregnancy. Exclusion criteria in the delivery room included abnormal labor progression and the use of analgesics.

Several tools were used for data collection include the demographic and fertility questionnaire and the Iranian Religious Coping Scale (IRCS). The IRCS designed by Aflakseir and Coleman. This scale consists of 22 items. Its scoring system is based on a 5-point Likert scale ranging from 0 (not at all) to 4 (a great deal). It also has five dimensions, including the practice of religious coping (6 items), benevolent reappraisal (6 items), negative religious coping (4 items), and passive and active religious coping (3 items each). This questionnaire does not have a total score. High scores on each dimension indicate high religious coping on that dimension. Cronbach's alpha of 0.89 was acceptable for the subscale of practice, 0.79 for benevolent reappraisal, 0.79 for negative religious coping, and 0.72 and 0.79 for passive and active religious coping, respectively [34].

The original Childbirth Self-Efficacy Inventory (CBSEI) designed by Lowe was another tool used for data collection [35]. In the present study, we used the Iranian version of CBSEI that has been psychometric by Khorsandi et al.. The Iranian Childbirth Self-Efficacy Inventory (ICBSEI) is a 36-item self-report tool with two subscales of outcome expectancy (OE) and efficacy expectancy (EE) (18 items on each subscale), as well as two total scores. The total score ranges from 18 to 180. Higher scores indicate higher levels of OE or EE for labor coping. Items are scored on a 10-point self-report scale ranging from one (not helpful at all) to ten (very helpful) for the OE subscale, and from one (not sure at all) to ten (very sure) for the EE subscale. Each subscale of the questionnaire has a high internal consistency of 0.88 for OE and 0.88 for EE [36].

The visual analog scale (VAS) for pain measurement was the last tool used in this study. The VAS scale has a 10 cm horizontal line grading from zero to 10. Zero typically represents “no pain at all,” whereas ten signifies the “worst pain” imaginable. VAS is one of the most commonly used measures of pain intensity in pain research, which has been repeatedly proven to be reliable in various studies [37, 38].

Sampling began at health centers after the approval of the project by the ethics committee of Iran University of Medical Sciences with the code (IR.IUMS.REC.1397.1007) and obtaining a license from Hormozgan University of Medical Sciences. Samples were recruited from health centers and followed up during the first stage of labor in the hospital for labor pain. First, the objectives of the study and the principle of confidentiality were explained to the eligible samples in the selected health centers, and then the researcher obtained informed written consent from the individuals and asked them to complete the demographic and fertility questionnaire, IRCS, and ICBSEI automatically. After completing the questionnaires, the researcher gave his phone number to the subjects so that they could inform the researcher when to visit the hospital for the NVD. When the subjects went to the hospital for delivery, the researcher was present in the delivery room and measured the pain intensity in dilatations of 5–7 cm and 8–10 cm after vaginal examination using the VAS ruler. Data analysis was performed on 200 samples because, at the time of the study, participants were referred to the hospital for delivery. Of those, 23 did not meet the inclusion criteria of the present study. Four participants had preterm labor. Two participants had neonatal over four kg. Two participants were bleeding. One participant was hospitalized due to reduced fetal movements. Four participants underwent cesarean sections due to abnormal progression of labor. Eight participants used spinal anesthesia to relieve labor pain, and two participants went to a private hospital for delivery. Data were analyzed by SPSS software version 22 using the independent *t*-test, one-way ANOVA, and the Pearson correlation test at a significant level of less than 0.05.

## 3. Results

The mean and standard deviation of participants' age was 23.32 ± 4.18. The mean and standard deviation of pain intensity in dilatation of 5–7 cm and 8–10 cm were 7.56 ± 2 and 8.89 ± 1.63, respectively. The mean and standard deviation of outcome expectancy and efficacy expectancy were 145.86 ± 25.47 and 131.72 ± 30.29, respectively. More information about the demographic and fertility characteristics of the subjects is present in Table 1.

The relationship between demographic and fertility variables includes age, woman's education, woman's occupation, economic status, abortion history, wanted and unwanted pregnancy, baby's gender, and participating in childbirth preparation classes with OE and EE were examined. Except for the significant relationship between abortion and OE, there was no statistically significant relationship between other variables and OE and EE.

The relationship between religious coping dimensions and pain intensity and childbirth self-efficacy subscales are presented in Tables 2 and 3.

## 4. Discussion

This study aimed to determine the relationship between religious coping, pain severity, and childbirth self-efficacy in primipara women. The results showed that with increasing benevolent reappraisal, the pain intensity decreased in dilatation of 5–7 cm, and with increasing negative religious coping, the pain intensity decreased in dilatation of 8–10 cm. Also, with increasing religious practices, benevolent reappraisal, and active religious coping, the outcome expectancy of pregnant women increased. But, with increasing negative religious coping, the outcome expectancy of pregnant women decreased. Efficacy expectancy also increased with increasing religious practices, while with increasing negative and passive religious coping, the efficacy expectancy decreased.

Taghizdeh et al. found that most participants in their study had a positive attitude towards labor pain. They considered labor pain as an opportunity for spiritual growth and excellence. This result is similar to the content of two items from the dimension of benevolent reappraisal in the present study, including “the hardships I endured brought me closer to God” and “I considered difficulties and hardships as a factor to strengthen my faith and transcendence of my soul” [4]. Religious coping and religious teachings have been considered a way to reduce anxiety, and since, in some cases, reducing anxiety reduces pain, they may also affect the severity of the pain [39]. The results of studies showed that reading the Quran verses affects the severity of labor pain [40, 41]. Esmaeili et al. and Ismaili et al. reported that reading the Qur'an by participants in their study not only reduced the severity of labor pains but also significantly shortened the duration of labor [40]. In the present study, the items of the Quran reading, prayer, remembrance, and supplication were all in the dimension of religious practices. Contrary to the findings of Esmaeili et al., there was no relationship between this dimension and the severity of pain in the two dilatations of 5–7 and 8–10 cm, which may be due to the time of the Quran reading. As in the mentioned studies, the Quran was read during the labor pain. While in the present study, it was read during pregnancy.

A review study examined the effect of religious-spiritual coping on pain intensity and reported that using religious coping could be a good way to control pain [42]. One of the ways that Folkman and Lazarus pointed out in problem-focused strategies is positive reappraisal [43]. This coping refers to spiritual growth and excellence in painful situations. Golchin showed positive reappraisal coping reduces pain, which is consistent with items in the dimension of benevolent religious coping of our study that had a significant direct relationship with pain intensity [44]. Mariza and Anggraini recommended religious coping to midwives as a way to manage the pain of the first stage of labor [45]. The results of another descriptive study, which examined the effect of spirituality on pregnancy, showed that spirituality increased self-confidence and reduced pain and anxiety during childbirth delivery [46]. Ghodrati and Ebrahimi also confirmed in their review study that religious-spiritual interventions, in addition to reducing stress, anxiety, and depression, reduce pain [47]. The results of the abovementioned studies are consistent with the results of the present study.

In the present study, with increasing negative religious coping, the pain intensity of participants in labor decreased, although the intensity of this relationship was weak. The dimension of negative religious coping items of the present study included forgetfulness, neglect by God, disappointment by God, and the feeling of anger towards God that is usually expressed. The results of a study showed that expressing emotions and being exposed to stressors decrease pain intensity and improve psychological symptoms [48]. Perhaps one of the factors that reduced pain intensity in this study was that the subjects were focusing less on the pain when they were expressing their anger and despair to God.

Consistent with the results of the present study, Vasigh et al. reported that spiritual health, religiosity, and religious coping can increase self-efficacy and pain tolerance in individuals [42].

Abdel et al. also reported that religiosity has a significant effect on self-efficacy and mental health [49]. The results of another study showed that religious coping is a factor in enhancing self-efficacy in people undergoing hemodialysis [50]. The results of these studies are consistent with the results of the present study. However, Woods et al., in a study to examine the relationship between religious-spiritual dependencies and self-efficacy in adolescents attending treatment clinics, found that such dependencies did not affect the self-efficacy of participants [51]. Perhaps the reason for the difference in the results of this study and the present study is the use of different study populations (adolescents in the abovementioned study). The norm crisis of adolescence affects religious and spiritual affiliations. Wood et al. have also used people of different religions (Christian, Muslim, Jewish, Hindu, atheist, etc.), while in the present study, only Muslims were included. Psychologists argue that positive religious coping helps a person understand the meaning of life events, especially painful events, and may empower people by creating support and vitality in their spirits [52]. The results of a study showed that increasing spiritual health in adolescents with thalassemia decreased their despair and increased their social empowerment [53]. Findings from a study in Italy also showed that spiritual care improves the health of cancer patients and improves their quality of life by increasing their ability [54].

## 5. Limitations

Since some religious coping varies according to the religion of the people of each country, we used a religious coping scale in the present study that was designed in Iran. Therefore, it can limit the generalizability of the results.

The questionnaires of the present study were self-administered, so the responses to some items might have been influenced by the personal characteristics, diagnosis, and mood of the subjects. It could have affected the outcome of the study, which was beyond the control of the researchers.

## 6. Conclusion

The results of the present study showed that with increasing some dimensions of positive religious coping, childbirth pain intensity and self-efficacy increase, and with increasing negative and passive religious coping, childbirth self-efficacy decreases. Attention to various ways of religious coping can provide a more comprehensive view for experts working with pregnant women so that they can design intervention programs to improve or enhance the quality of care of pregnant women.

## Figures and Tables

**Table 1:** Demographic and fertility characteristics of the participants (*n* = 200).

Characteristics	*N* (%)
Woman's education	
<High school	59 (29.5)
Diploma	85 (42.5)
Academic	56 (28)
Woman's occupation	
Housewife	185 (92.5)
Employed	15 (7.5)
Spousal's occupation	
Employee	34 (17)
Free	117 (58.7)
Worker	46 (23)
Unemployed	3 (1.5)
Economic status	
Favorable	38 (19)
Relatively favorable	125 (62.5)
Undesirable	37 (18.5)
Abortion history	
Yes	27 (13.5)
No	173 (86.5)
Gestational age	
32–35	87 (43.5)
36–39	80 (40)
≥40	33 (16.5)
Pregnancy	
Wanted	182 (91)
Unwanted	18 (9)
Wanted and unwanted baby's gender	
Wanted	182 (91)
Unwanted	18 (9)
Gender	
Boy	111 (55.5)
Girl	89 (44.5)
Participating in childbirth preparation classes	
Yes	28 (14)
No	172 (86)

**Table 2:** The relationships between religious coping and pain severity of the primiparous women (*n* = 200).

Dimensions of religious coping	Pain intensity in dilatations of 5–7 cm		Pain intensity in dilatations of 8–10 cm	
	*r*	*P* value	*r*	*P* value
Practice	−0/066	0.35	0.005	0.939
Benevolent reappraisal	−0.154	**0.029**	−0.024	0.731
Negative	0.094	0.186	−0.154	**0.030**
Active	−0.095	0.183	−0.013	0.851
Passive	−0.041	0.563	−0.046	0.520

**Table 3:** The relationships between religious coping and childbirth self-efficacy of the primiparous women (*n* = 200).

Dimensions of religious coping	Outcome expectancy		Efficacy expectancy	
	*r*	*P* value	*r*	*P* value
Practice	0.296	**<0.001**	0.236	**0.001**
Benevolent reappraisal	0.209	**0.003**	0.121	0.088
Negative	−0.204	**0.004**	−0.166	**0.019**
Active	0.238	**0.001**	0.128	0.071
Passive	−0.097	0.174	−0.152	**0.031**

## Data Availability

The data supporting this research article are available from the corresponding author on reasonable request.

## Supplementary Materials

Supplementary Materials STROBE checklist of information is in a supplemental file.
